# High Cost and Low Survival Rate in High Comorbidity Incident Elderly Hemodialysis Patients

**DOI:** 10.1371/journal.pone.0075318

**Published:** 2013-09-09

**Authors:** Yi-Ting Lin, Ping-Hsun Wu, Mei-Chuan Kuo, Ming-Yen Lin, Tzu-Chi Lee, Yi-Wen Chiu, Shang-Jyh Hwang, Hung-Chun Chen

**Affiliations:** 1 Department of Family Medicine, Kaohsiung Medical University Hospital, Kaohsiung Medical University, Kaohsiung, Taiwan; 2 Division of Nephrology, Department of Internal Medicine, Kaohsiung Medical University Hospital, Kaohsiung Medical University, Kaohsiung, Taiwan; 3 Department of Public Health, College of Medicine, Kaohsiung Medical University, Kaohsiung, Taiwan; 4 Department of Internal Medicine, College of Medicine, Kaohsiung Medical University, Kaohsiung, Taiwan; 5 Faculty of Renal Care, College of Medicine, Kaohsiung Medical University, Kaohsiung, Taiwan; University of Sao Paulo Medical School, Brazil

## Abstract

**Background:**

The comorbidity index is a predictor of mortality in dialysis patients but there are few reports for predicting elderly dialysis mortality and national population-based cost studies on elderly dialysis. The aim of this study was to evaluate the long-term mortality of incident elderly dialysis patients using the Deyo - Charlson comorbidity index (CCI) and to assess the inpatient and outpatient visits along with non-dialysis costs.

**Methods:**

Data were obtained from catastrophic illness registration of the Taiwan National Health Insurance Research Database. Incident elderly dialysis patients (age >75 years) receiving hemodialysis for more than 90 days between Jan 1, 1998, and dec 31, 2007, were included. Baseline comorbidities were determined one year prior to the first dialysis day according to *ICD-9 CM* codes. Survival time, mortality rate, hospitalization time, outpatient visit frequency, and costs were calculated for different age and CCI groups.

**Results:**

In 10,759 incident elderly hemodialysis patients, hazard ratios for all-cause mortality were significantly increased in the different age groups (*p* < 0.001) and CCI patients (*p* < 0.001). Death rates increased with both increasing age and CCI score. High comorbidity incident hemodialysis and elderly patients were found to have increased length of hospital stay and total hospitalization costs.

**Conclusions:**

This population-based cohort study indicated that both age and higher CCI values were predictors of survival in incident elderly hemodialysis. Increased costs and mortality rates were evident in the oldest patients and in those with high CCI scores. Conservative treatment might be considered in high comorbidity and low-survival rate end stage renal disease (ESRD) patients.

## Introduction

The demand for dialysis for elderly patients is increasing worldwide as a result of population aging [1] and age-related increases in chronic kidney disease incidence [2]. Data from the United States Renal Data System (USRDS) show that one in four patients starting dialysis in the United States (US) is over the age of 75 [3]. Similarly in Europe, the proportion of incident end stage renal disease (ESRD) in patients 65 years of age increased from 22% in 1980 to 55% in 2005 [4]. In addition, elderly patients are at greater risk for acute kidney injury [5,6], which is possibly related to the age-related increase in ESRD [7]. In Taiwan, the ESRD population is high and patients begin dialysis with very low residual renal function [8]. Taiwan is also one of the most rapidly ageing countries in the world. Individuals 65 years and older comprised > 7% of the total population in 1993 (based on the World Health Organization definition of an ageing country), and reached 9.01% in 2002, with an annual rate of increase of 0.2% [9].

For elderly ESRD patients, prompt renal replacement therapy is costly and does not necessarily extend survival [10,11]. Previous studies have also addressed cost-effectiveness of dialysis specifically [12]. Given the rapid growth of elderly populations, it is necessary to adopt practical and useful tool to make mortality prediction easier [13]. Several scales of comorbidity in dialysis patients have been validated, including the Charlson comorbidity index (CCI) [14], the index of co-existent diseases (ICED) [15], the Davies score [16], and the Wright-Khan indices [17]. The CCI is the most popular and has been validated in ESRD patients [18,19]. However, previous studies examining incident dialysis populations were performed in single centers and involved small numbers of patients. National population-based cost studies on elderly dialysis are also rarely discussed [20]. No population-based cohort study has compared the comorbidity index scores and hospitalization costs among incident elderly hemodialysis patients in Asian populations. The purpose of the present study is to predict long-term survival of incident elderly hemodialysis patients using the CCI in a nationwide cohort study and to evaluate the length of hospitalization and cost of treatment in this patient population.

## Materials and Methods

### Data Sources

We conducted a nationwide cohort using data from all elderly ESRD patients receiving hemodialysis in Taiwan’s National Health Insurance Research Database (NHIRD). The NHIRD has been previously described in detail [21]. The NHIRD established a registry system for catastrophic illnesses, including cancer, chronic mental illness, end-stage renal disease, and several autoimmune diseases. Outpatient and inpatient claims from beneficiaries with a catastrophic illness certificate are collected in the catastrophic illness profile and distributed as a package. For incident dialysis patients, dialysis catastrophic illness certificates are issued upon validation by 2 specialists, based on careful examination of the underlying disease, laboratory and imaging studies, and indications for dialysis treatment. Only individuals meeting the diagnostic criteria for major diseases are issued a catastrophic illness certificate. All incident hemodialysis patients over the age of 75 in the Registry of Catastrophic Illness Database were included. The NHI performs routine diagnostic validations by reviewing the original medical charts of all of the patients who applied for catastrophic illness registration. The database includes the following relevant information: diagnostic codes using the *International Classification of Disease, Ninth Revision, Clinical Modification* (ICD-9-CM) format, date of diagnosis, date of death, date of hemodialysis, date of every clinic visit, prescriptions, expenditure amounts, and outpatient/ inpatient claims for the beneficiaries with catastrophic illnesses during the period 1998–2007.

### Study Cohorts

Using the Registry for Catastrophic Illness Patient Database, we selected all patients diagnosed with ESRD defined as those who had catastrophic illness registration cards for ESRD (ICD-9 CM code 585) and who began hemodialysis more than 90 days prior to renal replacement therapy during the period of Jan 1, 1998, to Dec 31, 2007. The NHIRD began collecting statistics in 1997; however, our study cohort included data starting with 1998 to allow for a one-year wash-out period. In addition, we extended the observation time to 2008 in this cohort study. We included individuals older than 75 years of age who started hemodialysis in index days and excluded patients who received renal transplantation either before or after hemodialysis (n = 4).

The CCI was calculated to define the existing comorbidities using all diagnosis codes for the year prior to the index date on all inpatients and outpatients. Follow-up began on the index date and continued until death or the end of the study period if the patient was living (Dec, 31, 2008). The CCI is defined by Charlson et al [14] and the Deyo-Charlson comorbidity index and is based on ICD-9 codes in claims data. It has been widely used in the analyses of the impact of comorbidities on mortality [22]. All hemodialysis patients with diabetes are defined as having diabetes with end organ damage [21]. We categorized the CCI into four groups based on the following index scores: <3, 4-6, 7-9, >10. Hazard ratios of hemodialysis patient mortality in the four comorbidity index groups and different age groups were analyzed. Median survival years between >85 and < 85 years of age were evaluated in the different CCI group.

### Service Use and Costs

Taiwan’s NHI is a government-run, single-payer NHI scheme financed by a combination of premiums and taxes. It compensates public and private delivery systems predominantly through a fee-for-service basis. The NHI program allows patient freedom of choice when seeking medical care and uses cost-sharing strategies to reduce unnecessary services. An insured patient would incur additional cost in the form of payment for services not covered by the NHI as well as user fees and co-payments for NHI-covered services. User fees are levied for contact with the provider, whereas co-payments vary according to the type of provider. Co-payments are most expensive for outpatient care at medical centers and least expensive for clinics [23]. The medical care expenditure data used in the present study include medical expenditures of hospitalization services for the study subjects. All medical costs presented in the study were converted from Taiwan dollars ($NT) to US dollars using an exchange rate of 32.59:1, which was based on the average exchange rate from 2000 to 2006. Frequency and service costs were analyzed for inpatient services stratified by different age and CCI groups.

### Statistical Analysis

Data were summarized using proportions and mean values (±standard deviation) as appropriate. The association between the CCI or age groups and mortality was assessed using the Cox proportional hazards model and adjusting for age and the CCI, Kaplan–Meier estimates with log-rank tests were used to show survival in the follow-up. In the mortality analyses, the patients were followed until event (death) or censoring (lost to follow-up or end of the follow-up period), whichever occurred first. Analyses were performed using the SAS statistical package software (version 9.3; SAS Institute Inc, www.sas.com). All statistical tests were 2-sided with p<0.05 considered statistically significant.

## Results

The study population used for these analyses was representative of the Taiwan hemodialysis population and consisted of 10,759 incident elderly patients (age >75 years of age) with ESRD. Patients’ clinical characteristics and CCI scores are listed in Table 1. The cumulative prevalence of comorbid conditions in incident elderly ESRD patients before HD initiation is indicated in Figure S1. The patient mean age was 79.9 (± 3.9) years. In our study cohort, 46.3% of patients were women, and 46.7% were diabetic. Other major comorbidities in elderly patients included myocardial infarction(12.1%), congestive heart failure(55.7%), peripheral vascular disease(14.2%), cerebral vascular disease(49%), dementia(18.4%), neoplasia(21%), and metastatic malignancy(3.9%) (Figure S1).

**Table 1:** Basic demographics and characteristics of incident elderly hemodialysis patients.

N = 10,759	N, mean	%, SD
Age (years)	79.9	3.9
Age group		
75-79	6,342	59.0
80-84	3,210	29.8
85-89	1,000	9.3
>90	207	1.9
Gender		
Male	4,982	46.3
Female	5,777	53.7
Charlson comorbidity index		
<3(n %)	2,588	24.1
4-6	3,716	34.5
7-9	3,348	31.1
>10	1,107	10.3

**Figure 1:**
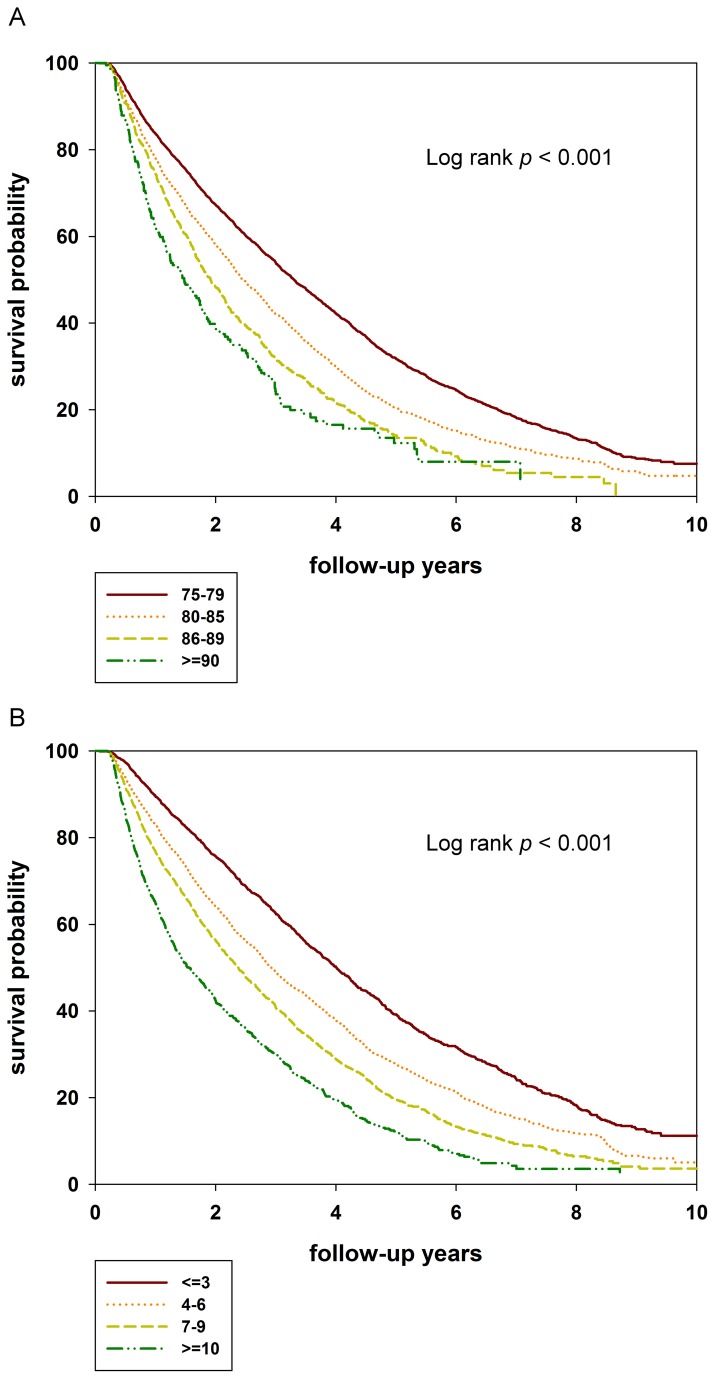
Survival curve stratified by age and Charlson comorbidity index (CCI) (A) Kaplan-Meier curves for 10 year survival by age. (B) Kaplan-Meier curves for 10 year survival by CCI. Survival calculations start 90 days after beginning dialysis. The survival rate of incident elderly hemodialysis patients declined as age and CCI increased.

After ranking subjects according to CCI scores, they were categorized into four groups. Based on cox proportional hazard models and age stratification, extremely elderly patients had significantly increased mortality compared with reference groups (75 to 79 years of age). For patients 80-84 years old, the adjusted hazard ratio (aHR) was 1.37 (95% confidence interval [CI], 1.30-1.44), at age 85 to 89 years, the aHR was 1.81 (95% CI, 1.67-1.96). Patients older than 90 years of age displayed an aHR value of 2.27 (95% CI, 1.94-2.66) (Table 2). Survival curves for age stratified into four groups are shown in Figure 1. The mortality rates between males and females were not significantly different (*p* = 0.197). With regard to CCI scores, mortality increased steadily with increased comorbidity for both unadjusted and adjusted models. Compared to the lowest comorbidity group (CCI <3), fully adjusted models of CCI 4-6, CCI 7-9, and CCI >10 displayed hazard ratios for mortality of 1.39 (95% CI 1.31-1.49), 1.83 (95% CI 1.71-1.95), and 2.64 (95% CI 2.43-2.87), respectively (Table 2).

**Table 2:** The effect of age and CCI on dialysis patients survival - using Cox regression analysis (univariate and multivariable).

Variable	Crude HR	95% CI	*p*-value	Adjusted HR	95% CI	*p*-value
Age group, years						
75-79	1 (Ref.)	-		1 (Ref.)	-	-
80-84	1.36	1.29-1.43	<0.001	1.37	1.30-1.44	<0.001
85-89	1.76	1.62-1.90	<0.001	1.81	1.67-1.96	<0.001
>90	2.18	1.87-2.55	<0.001	2.27	1.94-2.66	<0.001
Sex						
Men	1 (Ref.)			1.03	0.98-1.08	0.197
Women	1.01	0.96-1.05	0.829	1 (Ref.)	-	
CCI						
<3	1 (Ref.)	-		1 (Ref.)	-	
4-6	1.41	1.32-1.50	<0.001	1.39	1.31-1.49	<0.001
7-9	1.80	1.69-1.93	<0.001	1.83	1.71-1.95	<0.001
>10	2.60	2.39-2.83	<0.001	2.64	2.43-2.87	<0.001

Footnotes: CCI, Charlson comorbidity index; HR, hazard ratio; CI, confidence interval

**Figure 2:**
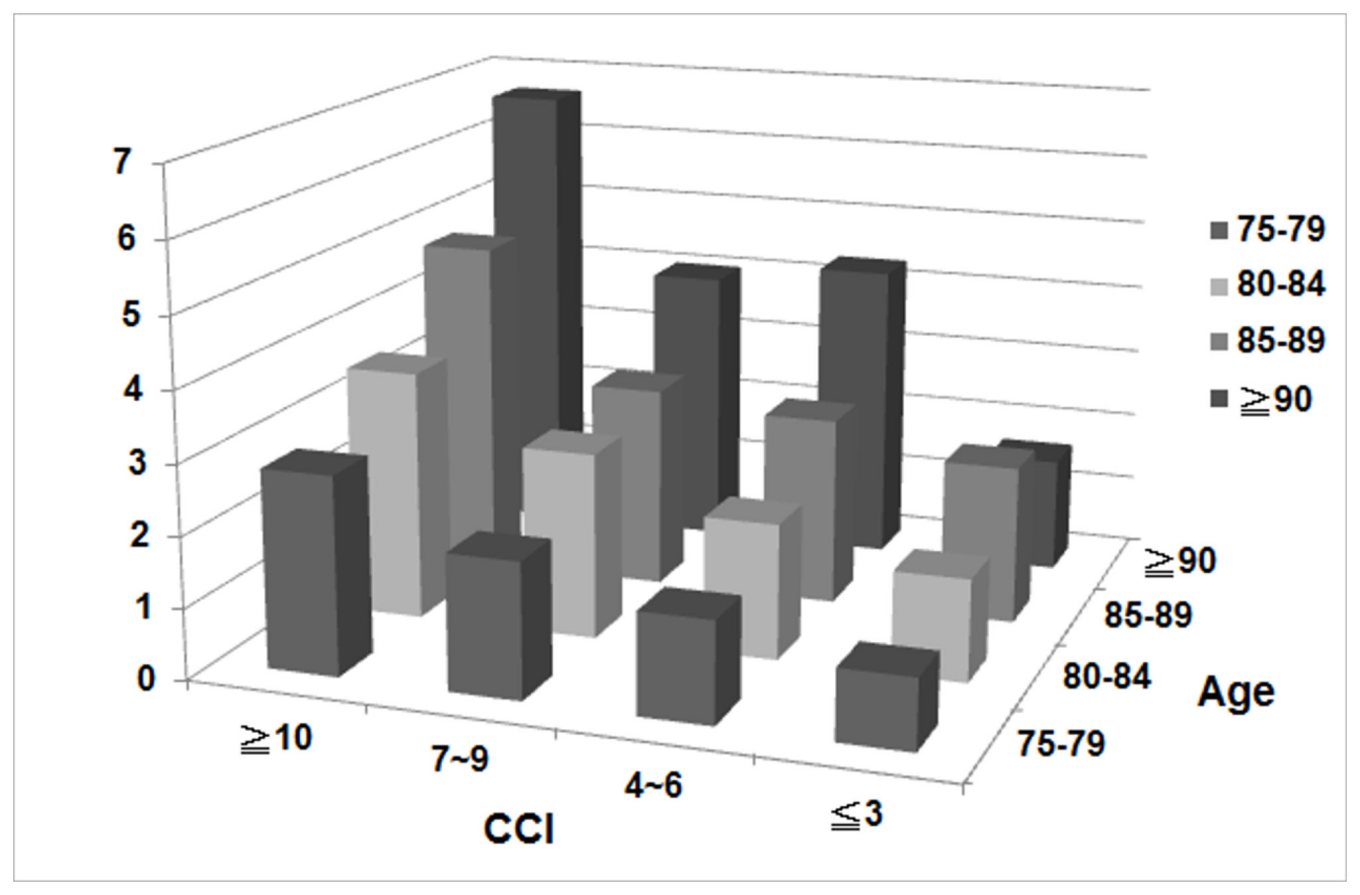
Increase mortality with increased age and CCI in incident elderly hemodialysis cohort. The bars represent hazard ratios by age group and CCI.

**Figure 3:**
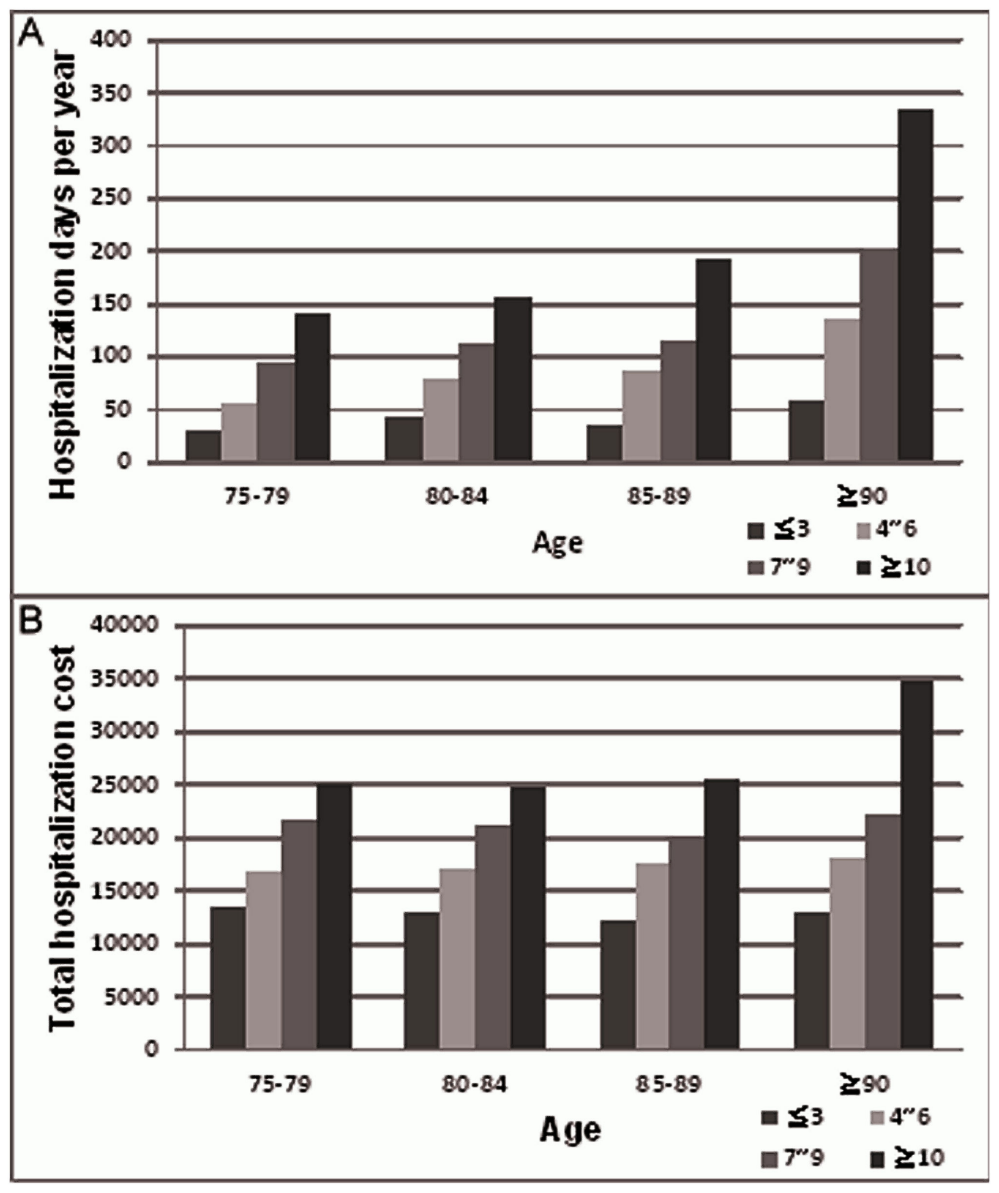
Hospitalization days per year and total hospitalization cost stratified by age and Charlson comorbidity index in elderly hemodialysis patients. (A) Increased duration of hospital stay for patients in the same age group with increased CCI levels (B) Increased mean admission costs (US dollars) for patients in the same age group with increased CCI levels (one United States dollars = 32.59 New Taiwan dollars).

Survival curves for CCI stratified according to the four comorbidity index groups are showed in Figure 1. Death rates increased with both increasing age and CCI score. The hazard ratios of mortality stratified according to age and CCI were significantly increased in elderly patients. greater than or equal to 90 years old with CCI 4-6, CCI 7-9, CCI > 10 and elderly patients 85-89 years old with CCI > 10 (Figure 2). Median years of survival steadily decreased with increased CCI scores. For patients > 85 years old with CCI > 10, the median survival was merely 1.04 years. In contrast, patients > 85 years old with CCI < 3 exhibited a median survival of 2.33 years (Table 3). In addition to the low survival rate of high CCI elderly patients, the duration of hospital stay was increased in this group (Figure 3A). As for the number of hospitalization days, patients 85-89 years of age with CCI > 10 experienced 193 hospital days, and patients > 90 years of age with CCI 7-9 experienced 203 hospital days. In incident hemodialysis patients > 90 years old and CCI > 10, the number of hospital days per year were 335 (Figure 3A). The hospitalization costs for elderly hemodialysis patients of the same age increased with increasing CCI (Figure 3B). The hospitalization costs for patients > 90 years old with CCI > 10 were thirty-four thousand nine hundred and seven US dollars and twenty-five thousand five hundred and eighty-four US dollars for patients 85-89 years old with CCI > 10.

**Table 3:** Median survival years in patients greater than or equal to 85 years old or < 85 years old with different Charlson comorbidity index values.

	Charlson comorbidity index			
	<3	4-6	7-9	>10
Median survival years in two age groups				
>85 years	2.33	1.90	1.67	1.04
< 85 years	4.24	3.14	2.47	1.63

## Discussion

CCI studies focusing on elderly patients starting hemodialysis are rare but have been previously documented previously. Risk stratification scores based on age, functional status, comorbidity, and planned versus unplanned dialysis initiation [24] or on body mass index, functional status, and early versus late referral [10] have been proposed for elderly patients. Because dialysis does not always enhance survival in elderly patients, distinguishing the patients with poor prognosis before dialysis is important. In this population-based study, the CCI was a predictor of survival in our study population. Moreover, the metric is easy to use and can instantly supply information that can be used by a clinician to make the decision to initiate hemodialysis. Our study demonstrated that an increased CCI was predictive of decreased long-term survival in incident elderly hemodialysis patients. In extremely elderly patients, an increased hazard ratio was evident in CCI groups (Table 2) and in patients with lower median survival years, especially patients older than 85 years of age (Table 3).

Previous studies have demonstrated that 1- and 5-year survival rates in incident dialysis patients over 75 years old were 53.5% and 2.4%, respectively [25]. In the North Thames Dialysis Study in the UK, the one-year survival for dialysis patients over 70 years of age was 71% [26]. A Canadian database study from the late 1990s found that patients older than 75 years of age exhibited 1- and 5-year survival rates of 69% and 20.3%, respectively, after hemodialysis initiation [27]. For those patients older than 90 years of age when dialysis was initiated, 1-year survival rates were <50% [28]. Similar results were found in the present study.

For patients older than 85 years with CCI > 10, median survival was only 1.04 years (Table 3). Survival time was dramatically decreased in patients with CCI > 10 regardless of age. A cohort study in the United Kingdom [29] showed that elderly patients who had dialysis when estimated glomerular filtration rate fell below 15 ml/min per 1.73 m^2^ could live longer than those who only received conservative treatments with the exception of dialysis. However, it is important to note that this difference was not observed in those patients with the highest comorbidities [30].

For patients over 65 years of age, the prognosis of dialysis treatment is similar to that of colorectal cancer [31]. In one series, hemodialysis was associated with poor outcome after cardiopulmonary resuscitation, and only 8% of dialysis patients receiving cardiopulmonary resuscitation survived to hospital discharge. Moreover, only 3% were still living 6 months later [32]. Additionally, dialysis in elderly populations is associated with increased hospitalization and complication rate. A previous study showed that elderly patients (>75 years) on hemodialysis spent 20% of their time in the hospital [25]. In the present study, patients older than 85 years of age with CCI >10 and patients older than 90 years of age with CCI 7-9 spent more than half of the year in the hospital.

It is controversial to determine whether patients with ESRD are suitable for dialysis because the medical and ethical decision is increasingly challenging in older patients [33]. However, for elderly incident dialysis patients with high comorbidities, factors to consider include poor quality of life, burden of life-sustaining treatment, conservative treatment, or palliative care [34]. Conservative care includes the multidisciplinary management of uremic symptoms through diet and medications, such as erythropoietin and diuretics, as well as psychosocial support and eventual palliative care. Maximum conservative management can provide a substantial increase in survival time and achieve similar numbers of hospital-free days compared with hemodialysis in elderly patients with high comorbidities [35]. Hence, conservative management is an alternative choice [36] and informed consent for elderly patients should note the risks, benefits, and burdens associated with dialysis along with the decline in functional status after dialysis [37].

The findings of our study reveal the useful information regarding real world data in the mortality rate, survival time, and length of hospital stay among incident elderly hemodialysis patients. Although estimates of life expectancy are crucial for determining the benefits of therapy, other important factors, such as patient preferences and expected quality of life should be considered. The decision-making process should begin early, and should be made on an individual basis. Both age and comorbidity need to be taken into account in developing policy development related to the provision of hemodialysis to elderly people with end-stage renal failure.

The NHI scheme covers dialysis expenses in the Taiwanese healthcare system. The high cost of dialysis therapy has raised questions concerning universal access to dialysis. The present study reveals an increase in the number of hospitalization days and costs for patients with higher CCI scores in the same age groups. Although not assessed, we surmise that these high comorbidities that patients experience increased non-medical costs and increased the need for family support given the frequent inpatient and outpatient visits. As revealed in the North Thames Dialysis Study (NTDS) Group publication, the majority of costs were allocated to dialysis treatment and transportation (70%), hospitalizations (12%) and medication (12%) [38]. The cost-effectiveness of dialysis services in elderly populations was studied previously [12,39] but the modality choice of hemodialysis or peritoneal dialysis remains controversial given cultural differences in Taiwan. In addition, elderly hemodialysis overtreatment and ethical dilemmas were evident [40] but the decision to provide dialysis or conservative treatment still needs to be balanced with medical comorbidities, quality of life, life expectancy, and patients’ wishes.

The present study using the NHI database has three strong points. First, it included a large number of patients free of selection bias because more than 99% of patients meeting study criteria were included in the database. Second, the nationwide database is comprehensive and unlikely to miss many hospital admissions and costs. Third, mortality data are also expected to be accurate. However, this study has several limitations. First, laboratory data and measurements of physical function were not available from the NHIRD. Although these clinical parameters may influence the mortality rate, the CCI still performed better than age, diabetes, cardiovascular disease, or albumin [41]. Second, certain parameters that may have improved the performance of our study population were not available in this database, such as information regarding dialysis access, dialysis dose, modality, residual renal function, and other treatment factors during follow-up. Third, patients who received dialysis < 90 days were excluded from this study because we wanted to eliminate the possibility of including patients with acute renal failure or renal failure patients with terminal illness. Finally, quality of life was not measured in the present study. In the very elderly, quality of life is perhaps more important than quantity. Quality of life is an important factor in determining treatment regiments in elderly patients. However, increased hospitalization days in extremely elderly patients with increased CCI could be indicative of poor quality of life.

In conclusion, age and CCI scoring are useful for predicting long-term mortality in incident elderly hemodialysis patients. Increased length of hospital stay, low survival rate, and increased utilization of national health insurance was observed in higher comorbidity index elderly dialysis patients. These findings help to clarify the risks and benefits of hemodialysis in elderly patient populations and are informative for dialysis decision-making process. Conservative treatment might be considered for ESRD patients with high comorbidities and low survival rates.

## Supporting Information

Figure S1 **Cumulative prevalence of comorbid conditions in incident elderly end-stage renal disease patients before hemodialysis initiation.**
(TIF)
