# Supplementary figures and images for: High Cost and Low Survival Rate in High Comorbidity Incident Elderly Hemodialysis Patients
Source: PLoS One. 2013 Sep 9;8(9):e75318. doi: 10.1371/journal.pone.0075318 (PMC3767633; doi:10.1371/journal.pone.0075318)

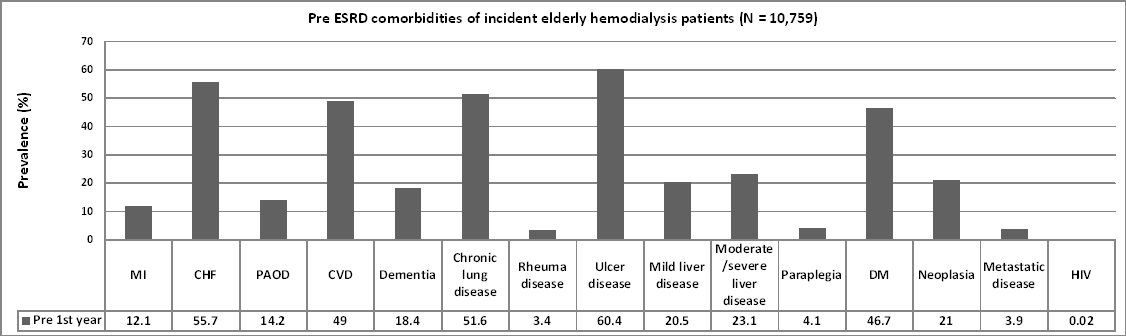

Supplement: Figure S1 — Cumulative prevalence of comorbid conditions in incident elderly end-stage renal disease patients before hemodialysis initiation. (TIF) [file pone.0075318.s001.tif]
